# Pregnancy Outcomes and Maternal Characteristics in Women with Pregestational and Gestational Diabetes: A Population-Based Study in Spain, 2016–2022

**DOI:** 10.3390/jcm13247740

**Published:** 2024-12-18

**Authors:** Ana López-de-Andrés, Rodrigo Jimenez-Garcia, David Carabantes-Alarcon, Natividad Cuadrado-Corrales, Andrés Bodas-Pinedo, Jesús Moreno-Sierra, Ana Jimenez-Sierra, José J. Zamorano-Leon

**Affiliations:** 1 Department of Public Health & Maternal and Child Health, Faculty of Pharmacy, Universidad Complutense de Madrid, 28040 Madrid, Spain; anailo04@ucm.es; 2 Department of Public Health & Maternal and Child Health, Faculty of Medicine, Universidad Complutense de Madrid, 28040 Madrid, Spain; dcaraban@ucm.es (D.C.-A.); mariancu@ucm.es (N.C.-C.); abodas@ucm.es (A.B.-P.); josejzam@ucm.es (J.J.Z.-L.); 3 Department of Surgery, Faculty of Medicine, Universidad Complutense de Madrid, 28040 Madrid, Spain; jesusmoreno@pdi.ucm.es; 4 Faculty of Medicine, Universidad San Pablo Ceu, 28668 Madrid, Spain; a.jimenez100@usp.ceu.es

**Keywords:** pregnancy, type 1 diabetes mellitus, type 2 diabetes mellitus, gestational diabetes, obstetric comorbidity index, severe maternal morbidity, hospitalizations

## Abstract

**Background/Objectives**: The objective of this study was to compare trends in the incidence of deliveries and in obstetric interventions and outcomes in women with and without type 1 diabetes (T1DM), type 2 diabetes (T2DM), and gestational diabetes (GDM). **Methods**: This was an observational study using the Spanish National Hospital Discharge Database (2016–2022). **Results**: A total of 1,995,953 deliveries were recorded between 2016 and 2022 (6495 mothers with T1DM, 5449 with T2DM, and 124,172 with GDM). The incidence of T1DM and GDM increased over time, although it remained stable in women with T2DM. Women with T2DM were more likely to have obstetric comorbid conditions (72.93%) than women with GDM (63.04%), women with T1DM (59.62%), and women who did not have diabetes (45.3%). Pre-eclampsia, previous cesarean delivery, and arterial hypertension were the most prevalent conditions in all types of diabetes. The highest frequency of cesarean delivery was recorded for women with T1DM (55.04%), followed by women with T2DM (44.94%), and those with GDM (28.13%). The probability of cesarean delivery was 2.38, 1.79, and 1.19 times greater for T1DM, T2DM, and GDM, respectively, than for women who did not have diabetes. The adjusted rate for severe maternal morbidity was significantly higher for women with T1DM (RR 2.31; 95%CI 2.02–2.63) and T2DM (RR 1.58; 95%CI 1.34–1.87) than for women without diabetes. **Conclusions**: The incidence of deliveries in women with T2DM remained unchanged between 2016 and 2022; the incidence of deliveries increased in women with T1DM and GDM. The prevalence of comorbidity and obstetric factors increased over time in women with T1DM and GDM.

## 1. Introduction

Diabetes in pregnancy is associated with a high risk of severe maternal and neonatal morbidity [1]. Gestational diabetes mellitus (GDM) increases the risk of fetal overgrowth, premature delivery, and type 2 diabetes mellitus (T2DM) [2]. A previous study conducted in Spain, from 2009 to 2015, reported that, in comparison with women who do not have diabetes, women with type 1 diabetes mellitus (T1DM) were twice as likely to have severe maternal comorbid conditions and more than three times more likely to have a preterm delivery. Furthermore, fetal overgrowth was also more than eight times more probable among women with T1DM [3]. Women with T2DM are more likely to have pre-existing hypertension [4]. Malaza et al. [5] recently observed that women with pregestational diabetes mellitus (PGDM) are at greater risk of adverse pregnancy outcomes than women with GDM [5].

As a reflection of the worldwide increase in the frequency of diabetes, the incidence of PGDM and GDM has increased constantly. Various studies show that GDM affects between 5.4% (95%CI 3.8–7.8%) and 10.9% (95%CI 10–11.8%) of pregnant women in developed countries [6,7,8]. In a study carried out in the USA, the incidence of PGDM increased significantly from 0.8 per 100 deliveries in 2000 to 1.6 per 100 deliveries in 2019 [9]. From 2010 to 2020, the total number of live births in Belgrade (Serbia) was 196,987, and the incidence of diabetes in pregnancy was 3.4%, with the total incidence of pre-gestational diabetes of 0.7% [10]. In Tuscany (Italy), from 2010 to 2018, among 206,917 singleton live births, GDM was diagnosed in 21,613 pregnancies (10.46%) and pregestational diabetes in 979 (0.47%) [11]. Data from Germany, for the period 2013–2019, showed that, among 4,991,275 singleton births, GDM was documented in 283,210 (5.7%) and pre-gestational in 46,605 (0.93%) cases [12].

Population-based studies carried out in Spain between 2001 and 2015 concluded that the number of deliveries in women with PGDM and GDM increased over time, with the more frequently associated conditions as follows: pre-eclampsia, smoking, hypertension, and obesity [3,13]. Updated national estimations for diabetes in pregnancy and associated complications (post 2015) could be important in terms of epidemiology and clinical practice and facilitate health care planning.

Given the current knowledge gap, the objective of the present study was to compare trends in the incidence of deliveries and obstetric interventions and outcomes between women with and without T1DM, T2DM, and GDM in Spain between 2016 and 2022.

## 2. Materials and Methods

### 2.1. Study Design and Data Source

We performed an observational retrospective population-based epidemiological study using the Minimum Basic Dataset for Hospital Discharges (Specialized Care Activity Registry [Spanish initials: RAE-CMBD]). This database contains information on individual patients admitted to Spanish public hospitals. It includes the patient’s sex, age, dates of admission and discharge, primary diagnosis, up to 20 secondary diagnoses, up to 20 procedures (therapeutic and diagnostic), and discharge destination (discharge, transfer to another health center, and death during admission) [14]. The information was coded according to the International Classification of Diseases, Tenth Revision (ICD-10). 

This study was carried out between 1 January 2016 and 31 December 2022. The study population comprised all women admitted for delivery based on the ICD-10 diagnostic and procedural codes used by Metcalfe et al. [15]. Admissions for delivery were stratified according to the diabetes status in any position of the diagnostic field in the RAE-CMBD as follows: women with T1DM, women with T2DM, women with GDM, and women without diabetes. The ICD-10 codes used to stratify the study population by diabetes status are shown in Table S1.

Universal screening for GDM in Spain is carried out in 2 stages, as set out in the national guidelines [16]. A glucose challenge test was performed with 75 g of glucose at weeks 24–28 of gestation, followed by an oral glucose tolerance test with 100 g if the result of the first test is positive (≥140 mg/dL). Diagnosis is confirmed by 2 plasma glucose values ≥105 mg/dL (fasting), 190 mg/dL (1 h), 165 mg/dL (2 h), and 145 mg/dL (3 h). In the case of women at high risk of GDM (age ≥ 35 years, obesity, personal or family history of diabetes, high-risk ethnic group), testing should be performed during the first trimester following the same approach. These recommendations have remained unchanged for the last few decades [17,18].

### 2.2. Study Variables

We used the obstetric comorbidity index (OCI) to identify comorbid conditions and obstetric factors in women with diabetes. The OCI is a weighted algorithm that assigns points according to the presence of pre-existing comorbid conditions, substance-related conditions, pregnancy-related conditions, and advanced maternal age (≥35 years). It was developed by Batteman et al. [19] and validated for use with the ICD-10 codes according to Metcalfe et al. [20]. 

The obstetric interventions studied were induced labor, cesarean delivery, forceps/vacuum extraction, and episiotomy. The ICD-10 codes used to identify these interventions are shown in Table S1.

Severe maternal morbidity was defined as the presence of at least 1 of 21 maternal morbidity indicators, as specified by Metcalfe et al. [15], using ICD-10 codes, including in-hospital maternal mortality, which was defined as the proportion of women who died during admission for delivery in each year of this study.

Prolonged maternal hospital stay was defined as a stay of more than 4 days after cesarean delivery and 2 days after a vaginal delivery, as reported by Metcalfe et al. [20]. 

Based on the ICD-10 codes shown in Table S1, we defined neonatal complications as preterm birth and fetal overgrowth. However, we were unable to analyze the presence of macrosomia, since, according to the methodology of the RAE-CMBD, this code can only be included as diagnostic code in the child’s discharge report but not in the mother’s report [3,13].

### 2.3. Statistical Methods

We calculated the incidence of admissions for delivery in women with T1DM, T2DM, and GDM per 10,000 deliveries.

We performed a descriptive statistical analysis by calculating percentages for categorical variables and means with standard deviation for continuous variables. The bivariate analysis was performed using the χ^2^ test, *t* test, and ANOVA, as applicable.

Analysis of the temporal trend in the frequency of pre-existing comorbidities before delivery and obstetric interventions performed was based on a nonparametric test for trends. The χ^2^ test was used to examine the association between diabetes status and neonatal morbidity and maternal morbidity.

Using multivariate logarithmic binomial models, we calculated the relative risk of obstetric interventions and severe maternal and neonatal complications for the different types of diabetes, as reported by Metcalfe et al. [21]. The analysis was performed using the OCI and women without diabetes as the reference category, adjusted for age. 

The statistical analysis was performed using Stata version 10.1 (Stata, College Station, TX, USA). Statistical significance was set at *p* < 0.05 (2-tailed).

### 2.4. Ethics

The Spanish Ministry of Health is the body responsible for administration of the database used in this study (RAE-CMBD). Access to the database is free upon request [22]. Given the anonymous and administrative nature of the database, and, in line with Spanish legislation, the individual patient’s written consent is not necessary on admission to the hospital. Similarly, ethics committee approval is not necessary.

## 3. Results

Between 2016 and 2022 in Spain, a total of 1,995,953 deliveries were recorded in public hospitals; of these, 6495 were coded in women with T1DM, 5449 in women with T2DM, and 124,172 in women with GDM. 

### 3.1. Trends in the Incidence of Deliveries in Spain Between 2016 and 2022

During the study period, the incidence rate per 10,000 deliveries increased significantly in women with T1DM (29.87 in 2016 vs. 35.12 in 2022; *p* = 0.001) and in women with GDM (429.95 in 2016 vs. 702.94 in 2022; *p* < 0.001), whereas it decreased in women without diabetes (9518.61 in 2016 vs. 9228.35 in 2022; *p* < 0.001). Among women with T2DM, the incidence of deliveries remained unchanged at around 27.3 per 10,000 deliveries, as shown in Table 1.

### 3.2. Trends in Maternal Age in Spain Between 2016 and 2022

Mean age was 35.22 years (SD: 5.31) in women with T2DM, 34.49 years (SD: 5.28) in women with GDM, 32.65 years (SD: 5.52) in women with T1DM, and 31.93 years (SD: 5.81) in women without diabetes.

The mean age of women admitted for delivery increased significantly in those with T1DM (32.35 in 2016 vs. 33.15 in 2022; *p* = 0.004) and in those who did not have diabetes (31.82 in 2016 vs. 32.11 in 2022; *p* < 0.001), remaining unchanged among those with T2DM and GDM, as shown in Table 1.

### 3.3. Comorbid Conditions and Obstetric Factors During Delivery 

As shown in Table 2, 72.93% of women with T2DM, 63.04% of women with GDM, and 59.62% of women with T1DM had at least one condition included in the OCI, compared with 45.3% of nondiabetic women.

The most prevalent obstetric factor was previous cesarean delivery, with values of 20.52% in women with T2DM, 18.38% in women with T1DM, 12.46% in women with GDM, and 8.71% in women without diabetes. 

The prevalence of comorbid conditions varied depending on the type of diabetes. Among those with T2DM, the prevalence of pre-existing hypertension was almost five times higher than among women with GDM. 

When comparing the four groups of women two by two, we found that the prevalence of mild/unspecified pre-eclampsia and severe pre-eclampsia, was significantly different between all groups. The same was found for gestational hypertension, with an exception made for T1DM versus T2DM. The highest differences were observed in the prevalence of mild and severe pre-eclampsia between women with T1DM and those with GDM (11.44% and 10.81% vs. 3.64% and 3.28%, respectively), as shown in Table 2.

Regarding placenta previa, the prevalence was only significantly different between women without and women with GDM (0.55% vs. 0.63%; *p* = 0.002)

**Figure 1:**
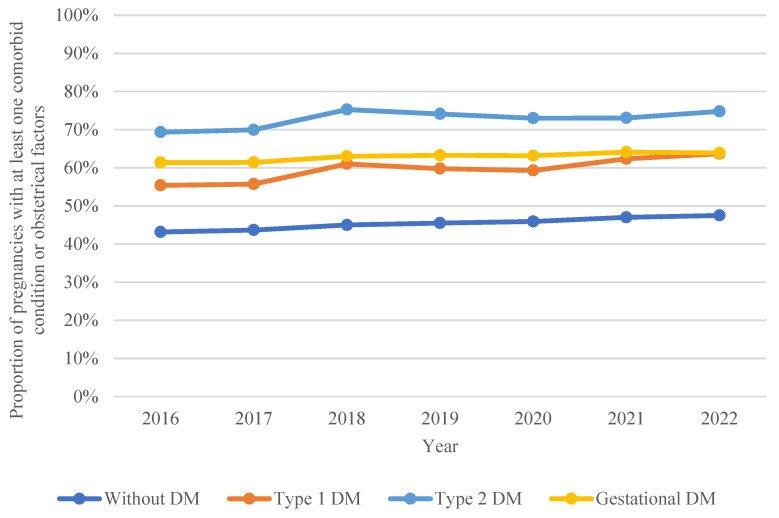
Temporal trends in the proportion of pregnancies in women with type 1, type 2, and gestational diabetes with at least one comorbid condition (excluding diabetes) compared with women without diabetes.

The OCI increased significantly throughout the study period among women with T1DM (55.40% in 2016 vs. 63.69% in 2022; *p* < 0.001), women with GDM (61.39% in 2016 vs. 63.91% in 2022; *p* < 0.001), women without diabetes (43.16% in 2016 vs. 47.51% in 2022; *p* < 0.001), and women with T2DM (69.34% in 2016 vs. 74.80% in 2022; *p* < 0.001). The prevalence of comorbid conditions according to the OCI was greater in women with T2DM than in those with other types of diabetes during each year of this study, as shown in Figure 1. 

### 3.4. Impact of Type of Diabetes on Obstetric Interventions and Maternal and Neonatal Health Outcomes

Induced labor was coded in 37.52% of women with T1DM, 37.36% of women with T2DM, and 36.7% of women with GDM. After adjusting for age and the OCI in the multivariate models and using women without diabetes as the reference category, the probability of induced labor was 1.33 times higher for women with T1DM and T2DM and 1.36 times higher for women with GDM, as shown in Table 3.

The frequency of induced labor increased significantly over time for all types of diabetes (*p* < 0.001), as shown in Figure S1.

Cesarean delivery was more common in women with T1DM (55.04%), followed by those with T2DM (44.94%) and those with GDM (28.13%). After the multivariate adjustment, the probability of a cesarean delivery was 2.38, 1.79, and 1.19 times greater for T1DM, T2DM, and GDM, respectively, than for women without diabetes (Table 3). However, the frequency of cesarean delivery remained constant in women with T1DM (*p* = 0.193) and T2DM (*p* = 0.573), although it increased from 28.57% to 29.35% in women with GDM (*p* < 0.001), as indicated in Figure S2.

Forceps and vacuum extractions were equally frequent in women with T1DM and those without diabetes but significantly less frequent in those with T2DM and GDM, whereas episiotomy was performed in a significantly lower percentage of women with diabetes than in those without diabetes, as shown in Table 3. 

The frequency of forceps and vacuum extraction fell significantly over time in women with T2DM and GDM (*p* = 0.029 and *p* < 0.001, respectively), as shown in Supplementary Figure S3. In the case of episiotomy, the frequency decreased significantly with time for all classes of diabetes (*p* < 0.001), as shown in Figure S4.

Rates of maternal morbidity and neonatal morbidity (preterm birth and fetal overgrowth) were highest for women with T1DM, followed by women with T2DM, GDM, and, finally, those without diabetes (Table 3). 

The adjusted relative risk for severe maternal morbidity was significantly higher in T1DM (RR 2.31; 95%CI 2.02–2.63) and T2DM (RR 1.58; 95%CI 1.34–1.87) than in women without diabetes. GDM did not increase the probability of severe maternal morbidity after adjusting for the OCI and age.

Preterm birth was 2.54 times more likely in women with T1DM, and fetal overgrowth 7.61 times more likely than in women without diabetes. These values were 1.81 and 4.71 for T2DM and 1.25 and 2.79 for GDM, respectively.

No significant changes in severe maternal morbidity over time were observed between women with diabetes (Figure S5). The frequency of preterm birth increased significantly only among women with GDM, increasing from 6.19% in 2016 to 6.99% in 2022 (*p* < 0.001) (Figure S6). The frequency of fetal overgrowth increased significantly among women with T1DM and among women with GDM (10.03% and 3.27% in 2016 vs. 13.85% and 5.25%, respectively; all *p* < 0.001) (Figure S7).

The risk of a prolonged maternal hospital stay was 2.54 times greater (95%CI: 2.44–2.65) for women with T1DM, 1.81 times greater (95%CI: 1.71–1.91) for those with T2DM, and 1.25 times greater (95%CI: 1.23–1.27) for those with GDM than for women without diabetes. Throughout the study period, the frequency of prolonged stay fell in women with T1DM (37.84% in 2016 vs. 28.79% in 2022; *p* < 0.001) and in women with GDM (17.50% vs. 16.19%; *p* < 0.001), remaining unchanged in women with T2DM (Figure S8).

## 4. Discussion

This nationwide study based on diabetes status in almost two million deliveries in Spain between 2016 and 2022 revealed a series of noteworthy findings. First, the temporal pattern in the incidence of GDM and PGDM is variable, with an increase in the incidence of GDM and T1DM during pregnancy and no change in the incidence of T2DM. Second, comorbidity and obstetric factors are more frequent in women with T2DM, remaining stable over time, although frequency increased in women with GDM and T1DM. Third, severe maternal comorbidity and preterm delivery were more common in women with T1DM and T2DM than in women who did not have diabetes. Fourth, the frequency of preterm birth and fetal overgrowth increased in women with GDM during the study period.

The results of the present analysis show that the rising trends detected in previous studies of administrative data [3,13] continued throughout 2022, except for the incidence of deliveries in women with T2DM, which remained stable. These results support findings from other population-based studies in which the incidence of GDM was shown to be increasing among pregnant women [4,9,11,23,24,25,26]. Consistent with previous data, advanced maternal age was associated with an increase in the incidence of GDM. In addition, the increased frequency of deliveries in women aged ≥35 years may contribute to these trends [3,24,26]. The specific differences detected for age underline the importance of personalized detection strategies and interventions, especially among older pregnant women [9]. Furthermore, this rising trend may be associated with an increase in the detection rate and the fact that high-risk pregnancies are increasingly frequent, for example, in obese women [24]. In our study, we can assume that the most relevant factor was the increase in the frequency of overweight and obesity. In this sense, there is robust evidence that the prevalence of overweight and obesity among women of childbearing age increased considerably during the study period in Spain [27].

Data on trends in PGDM from population-based studies are contradictory. In a study performed in the USA between 2000 and 2019, Nwachukwu et al. [9] concluded that the rate of PGDM remained stable between 2000 and 2009 (1.1 per 100 deliveries), although it increased gradually to 1.6 per 100 deliveries in 2019. However, data from a study performed in Canada between 2005 and 2018 indicate that the delivery rate among women with T2DM increased from 2.6 to 6.4 per 1000 deliveries (*p* < 0.0001), whereas that of women with T1DM remained unchanged at 3.0 per 1000 deliveries per year (*p* = 0.4301) [28]. In Italy, Gualdani et al. [11] reported similar data on delivery rates for women with T1DM, although the authors observed a significant decrease with respect to the incidence of T2DM. In the USA, Gorsch et al. [26] reported that, from the year 2000 to the year 2019, the rates of deliveries in women with T2DM increased from 1.8 to 7.3 per 1000 deliveries, remaining constant in women with T1DM (2.7 in 2000 to 2.8 per 1000 deliveries in 2019). In our study, the incidence of deliveries increased in women with T1DM and remained unchanged in women with T2DM. One possible explanation would be maternal age, which increased in women with T1DM and remained constant in those with T2DM. While the incidence of PGDM may not have increased as significantly as that of GDM, it continues to be a relevant cause for concern owing to the specific challenges it raises during pregnancy. The appropriate preconception care and effective management of PDGM are essential if we are to reduce the frequency of adverse outcomes both for the mother and for the neonate.

We highlight the main differences in comorbidity and factors that affect obstetric management and pregnancy outcomes according to the type of diabetes. Previous cesarean delivery was the most common factor in women with PGDM and in those with GDM. Outcomes in women with T2DM are poorer than in the other study groups. Women with T2DM more frequently had pre-eclampsia and pre-existing hypertension. One possible explanation for this finding could be that T2DM continues to be considered a less severe condition. The results of a UK study indicate that few women with T2DM were taking insulin (18%) or folic acid (5 mg) (22%) at the start of their pregnancy. However, almost two-thirds of women with T2DM (65%) were taking metformin, indicating a commitment to medical care yet revealing missed opportunities to improve preparation for pregnancy [29].

As was to be expected, the probability of induced labor was 30% higher in women with diabetes than in those without diabetes. This figure increased gradually throughout the study period. In the USA, induced labor has been associated with a reduction in the probability of neonatal mortality (*p* < 0.05) [30].

In our study, women with diabetes were more likely to undergo cesarean delivery than those without diabetes. Similar results were observed in other studies, where the risk for cesarean delivery was reported to be greater in women with PGDM and in women with GDM. Reischer et al. [31] recently found that pre-eclampsia and PGDM were independent factors associated with cesarean delivery (OR, 4.35 [95%CI 1.51–12.57] and 4.76 [95%CI 2.30–9.84], respectively). Karkia et al. [32,33] reported a 2.5-fold and 1.7-fold increase in the risk of cesarean delivery in 509 pregnant women with PGDM and 2089 pregnant women with GDM compared with 49,122 pregnant women without diabetes. Moreover, the lower number of episiotomies in women with T1DM, T2DM, and GDM compared with women without diabetes reflects a greater incidence of preterm deliveries and a greater incidence of cesarean delivery.

We found that the frequency of preterm births was significantly higher among women with T1DM and T2DM than in women without diabetes. In line with our findings, a population-based study performed in Sweden between 1998 and 2016 found that around 25% of women with T1DM gave birth prematurely [34]. While the reasons underlying this risk are not clear, it seems that insufficient hemoglobin A1c (HbA1c) control at the onset of pregnancy may reflect, in part, an increase in the incidence of obesity and associated insulin resistance [35]. However, premature birth rates also increase in women whose HbA1c falls within the reference range and in women with micro-or macroangiopathy or pre-eclampsia [36]. In our study, the prevalence of severe pre-eclampsia in women with T1DM was 10.81%; this may have contributed to the risk of preterm births of 3.88 (95%CI 3.68–4.10).

As was to be expected, severe neonatal and maternal morbidity was more common in women with PGDM, followed by women with GDM. We found that women with T1DM had higher values for the risk of severe maternal morbidity (adjusted RR 2.54; 95%CI 2.44–2.65). In the USA, Battarbee et al. [37] reported that newborns of mothers with PGDM had a more than 2-fold risk (RR 2.27; 95%CI 1.95–2.64) than nondiabetic mothers and a 1.96-fold greater risk (RR 1.96; 95%CI 1.63–2.35) than women with GDM. A study performed in Germany between 2013 and 2019 concluded that PGDM increased the risk of stillbirth (RR 2.34; 95%CI 2.11–2.59), whereas GDM was associated with a lower risk (RR 0.67; 95%CI 0.62–0.72) [23]. Despite these findings, our study did not reveal a significant change in severe maternal morbidity over time in the different groups.

Consistent with other authors [26], we found the risk of fetal overgrowth to be greater in women with T1DM. In a study of 180 pregnant women with T1DM, Morrens et al. [38] found a greater frequency of macrosomia in 42.5% of cases and correlated this finding both with the increase in weight and with HbA1c values during the early stages of pregnancy and, at delivery, possibly because of the long duration of diabetes or potentially unstable glycemia [38]. The above-mentioned complications once again highlight the critical importance of strict blood sugar control and the thorough management of women with PGDM, right from the early stages of pregnancy [39]. However, our study showed a significant increase in preterm birth and fetal overgrowth in women with GDM, possibly in association with excess pregestational weight, excessive weight gain, and advanced maternal age [40,41,42].

The main strength of our study lies in the use of the RAE-CMBD, an extensive database of hospital registries from the Spanish population covering a period of 7 years. The methodology applied has been addressed in detail elsewhere, thus reinforcing the credibility and reproducibility of our findings [3,13]. Of note, the RAE-CMBD includes almost all admissions to public hospitals in Spain (>95%) and enabled us to analyze trends and stratify results according to the presence of T1DM, T2DM, and GDM. Despite these strengths, our study is subject to a series of limitations. First, a significant limitation stems from the nature of the RAE-CMBD itself as an administrative database. Although useful for epidemiological research, it does not include important clinical aspects of the DM, for example, whether the patient is receiving insulin or other antidiabetic drugs, the duration of the disease, the degree of control obtained for DM and other cardiovascular risk factors, or the history of micro or macrovascular complications. Second, the indications for cesarean section, or whether it was urgent or elective, are not collected by the dataset so it cannot be determined to which point these issues are related to diabetic conditions. Third, regarding GDM, we lack the information on whether insulin was used or not for its treatment as this is not collected in the RAE-CMBD. This is relevant since previous studies have documented significant differences related to perinatal, neonatal, as well as pregnancy outcomes between those who required insulin or not [43,44,45]. 

Fourth, as commented before, complications that may occur in the newborn are coded in the child’s discharge report, and it is not possible, for reasons of confidentiality, to link the mother’s data with those of her child. Therefore, we could not analyze neonatal complications related to GDM such as shoulder dystocia or neonatal hypoglycemia. Fifth, regarding short-term maternal obstetric complications, we decided to use the 21 maternal morbidity indicators specified by Metcalfe et al. [15], using ICD-10 codes, in order to make our results comparable to other studies with the same standardized methodology, even if some important complications, such as polyhydramnios, oligohydramnios, intrauterine growth restrictions, or those after obstetric interventions as forceps, were not analyzed. Sixth, in the database, there is no recorded information on conditions previous to the hospitalization, such as fertility issues, in vitro fertilization, cases of recurrent pregnancy losses, etc., that might have influenced the outcomes and are known obstetric factors that may be associated with diabetes. Seventh, the RAE-CMDB only collects information on diagnosis and procedures during the hospital admission, so it is not possible to analyze the long-time maternal complications.

Finally, as in similar retrospective and observational studies that depend on the ICD codes obtained from large databases, our research could be susceptible to low sensitivity and specificity. This limitation could depend on the accuracy of the clinician when coding hospital diagnoses and procedures, which would in turn affect data quality. Nevertheless, previous studies performed in Spain and elsewhere have shown the validity of diagnosing diabetes and GDM using ICD codes in administrative health databases, as opposed to clinical registers. These studies concluded that the abovementioned databases can be used to address research questions effectively [46,47]. Spanish studies evaluating the validity of diagnosing diabetes using the RAE-CMBD reported a specificity of 97% (that is, almost all patients with a diabetes code really have the disease), a sensitivity of 55–63.7% (that is, the condition is not coded in some patients who really do have diabetes), and a kappa index of 0.6–0.7 [48]. Moreover, a systematic review from the USA reported that GDM (sensitivity, 71.0–81.3%; specificity, 99.4–99.6%; positive predictive value, 50.0–88.8%) and pre-existing diabetes (sensitivity, 78.0–95.3%; specificity, 99.4–100.0%; positive predictive value, 94.0–97.6%) were accurately coded on birth certificates and data for hospital discharges [49]. Similar values were recorded from Canadian databases [50]. Finally, this study did not include deliveries at private hospitals, which account for around 17% of all deliveries and where clinical characteristics and procedures may differ from those recorded in the National Health System [51].

## 5. Conclusions

In conclusion, the incidence of deliveries among women with T2DM has remained unchanged since 2016 but has increased among women with T1DM and GDM. The prevalence of comorbid conditions and obstetric factors increased over time in women with T1DM and GDM. During the study period, we observed an increase in the frequency of preterm deliveries in women with GDM, with the risk higher in women with T1DM and T2DM. All these findings highlight the need for more rigorous monitoring and specific interventions aimed at women with diabetes in order to reduce the risks associated with maternal morbidity and improve perinatal outcomes.

## Figures and Tables

**Table 1:** Incidence rate and demographic characteristics of delivery admissions in Spain, 2016–2022.

		2016	2017	2018	2019	2020	2021	2022	*p*-Value
Without DM	N	292,190	287,088	273,990	269,660	252,251	243,678	240,980	
	Rate per 10,000 deliveries	9518.61	9470.41	9303.38	9275.85	9218.96	9165.17	9228.35	<0.001
	Maternal age, mean (SD)	31.82 (5.66)	31.85 (5.76)	31.87 (5.82)	31.89 (5.86)	31.91 (5.88)	32.12 (5.81)	32.11 (5.89)	<0.001
Type 1 DM	N	917	933	975	960	887	906	917	
	Rate per 10,000 deliveries	29.87	30.78	33.11	33.02	32.42	34.08	35.12	0.001
	Maternal age, mean (SD)	32.35 (5.49)	32.29 (5.42)	32.99 (5.2)	32.56 (5.5)	32.5 (5.74)	32.7 (5.72)	33.15 (5.55)	0.004
Type 2 DM	N	662	712	724	831	763	880	877	
	Rate per 10,000 deliveries	21.57	23.49	24.58	28.58	27.89	33.1	33.58	0.176
	Maternal age, mean (SD)	35.04 (5.15)	34.72 (5.19)	35.15 (5.23)	35.27 (5.33)	35.48 (5.49)	35.21 (5.36)	35.55 (5.37)	0.054
Gestational DM	N	13,198	14,409	18,817	19,261	19,721	20,410	18,356	
	Rate per 10,000 deliveries	429.95	475.32	638.93	662.55	720.74	767.66	702.94	<0.001
	Maternal age, mean (SD)	34.43 (5.09)	34.51 (5.11)	34.49 (5.21)	34.47 (5.35)	34.44 (5.33)	34.55 (5.33)	34.54 (5.41)	0.189

DM, diabetes mellitus. *p*-value for time trend.

**Table 2:** Types of preexisting comorbidities and obstetrical factors in the current pregnancy as defined by the obstetrical comorbidity index.

	Weight *	Without DM (n = 1,859,837)	Type 1 DM (n = 6495)	Type 2 DM (n = 5449)	Gestational DM (n = 124,172)	*p*
Maternal Age, mean (SD)		31.93 (5.81)	32.65 (5.52)	35.22 (5.31)	34.49 (5.28)	<0.001
<20 years, n (%)	-	47,343 (2.55)	98 (1.51)	23 (0.42)	581 (0.47)	<0.001
20–34 years, n (%)	-	1,148,300 (61.74)	3834 (59.03)	2275 (41.75)	57,855 (46.59)	
35–39 years, n (%)	1	512,407 (27.55)	1953 (30.07)	2024 (37.14)	44,923 (36.18)	
40–44 years, n (%)	2	141,065 (7.58)	571 (8.79)	985 (18.08)	18,649 (15.02)	
≥44 years, n (%)	3	10,722 (0.58)	39 (0.6)	142 (2.61)	2164 (1.74)	
Alcohol Abuse, n (%)	1	1054 (0.06)	7 (0.11)	7 (0.13)	51 (0.04)	0.004
Asthma, n (%)	1	40,722 (2.19)	137 (2.11)	176 (3.23)	3108 (2.5)	<0.001
Cardiac Valvular Disease, n (%)	2	1823 (0.1)	11 (0.17)	8 (0.15)	154 (0.12)	0.007
Chronic Congestive Heart Failure, n (%)	5	320 (0.02)	15 (0.23)	7 (0.13)	26 (0.02)	<0.001
Chronic Ischemic Heart Disease, n (%)	3	192 (0.01)	4 (0.06)	9 (0.17)	17 (0.01)	<0.001
Chronic Renal Disease, n (%)	1	755 (0.04)	58 (0.89)	14 (0.26)	60 (0.05)	<0.001
Congenital Heart Disease, n (%)	4	1394 (0.07)	9 (0.14)	5 (0.09)	98 (0.08)	0.274
Drug Abuse, n (%)	2	5535 (0.3)	24 (0.37)	15 (0.28)	186 (0.15)	<0.001
Gestational Hypertension, n (%)	1	26,126 (1.4)	291 (4.48)	235 (4.31)	3514 (2.83)	<0.001
Human Immunodeficiency Virus, n (%)	2	1997 (0.11)	0 (0)	7 (0.13)	108 (0.09)	0.008
Mild/Unspecified Pre-Eclampsia, n (%)	2	38,130 (2.05)	743 (11.44)	470 (8.63)	4526 (3.64)	<0.001
Multiple Gestation, n (%)	2	39,211 (2.11)	142 (2.19)	120 (2.2)	3690 (2.97)	<0.001
Placenta Previa, n (%)	2	10,148 (0.55)	38 (0.59)	30 (0.55)	780 (0.63)	0.002
Pre-Existing Hypertension, n (%)	1	16,332 (0.88)	280 (4.31)	700 (12.85)	3494 (2.81)	<0.001
Previous Cesarean Delivery, n (%)	1	161,940 (8.71)	1194 (18.38)	1118 (20.52)	15,473 (12.46)	<0.001
Pulmonary Hypertension, n (%)	4	121 (0.01)	2 (0.03)	1 (0.02)	8 (0.01)	0.075
Severe Pre-Eclampsia, n (%)	5	36,445 (1.96)	702 (10.81)	374 (6.86)	4074 (3.28)	<0.001
Sickle Cell Disease, n (%)	3	7272 (0.39)	29 (0.45)	32 (0.59)	555 (0.45)	0.002
Systemic Lupus Erythematosus	2	1712 (0.09)	5 (0.08)	4 (0.07)	77 (0.06)	0.008
Obstetric Comorbidity Index, mean (SD)		0.8 (1.36)	1.67 (2.46)	1.84 (2.22)	1.26 (1.68)	<0.001
Obstetric Comorbidity Index, n (%)		842,478 (45.3)	3872 (59.62)	3974 (72.93)	78,274 (63.04)	<0.001

* Weight refers to the relative weight of each condition according to the obstetric comorbidity index derived by Bateman et al. [19]. The validation of this index with the ICD-10 codes was conducted by Metcalfe et al. [20]. *p*: χ^2^ test or ANOVA.

**Table 3:** Impact of diabetes mellitus types on obstetrical interventions and maternal and neonatal health outcomes.

	Without DM	Type 1 DM	Type 2 DM	Gestational DM
Induced Labor, n (%)	507,746 (27.3)	2437 (37.52)	2036 (37.36)	45,574 (36.7)
Crude RR (95%CI)		1.37 (1.32–1.43)	1.37 (1.31–1.43)	1.34 (1.33–1.36)
Adjusted RR (95%CI)		1.36 (1.31–1.42)	1.35 (1.29–1.42)	1.33 (1.32–1.35)
Cesarean Delivery, n (%)	390,507 (21.00)	3575 (55.04)	2449 (44.94)	34,925 (28.13)
Crude RR (95%CI)		2.62 (2.54–2.71)	2.14 (2.06–2.23)	1.34 (1.32–1.35)
Adjusted RR (95%CI)		2.38 (2.30–2.47)	1.79 (1.73–1.87)	1.19 (1.18–1.21)
Forceps/Vacuum Extraction, n (%)	219,998 (11.83)	725 (11.16)	432 (7.93)	14,082 (11.34)
Crude RR (95%CI)		0.94 (0.87–1.01)	0.67 (0.61–0.74)	0.96 (0.94–0.98)
Adjusted RR (95%CI)		0.95 (0.88–1.02)	0.68 (0.62–0.75)	0.94 (0.92–0.95)
Episiotomy, n (%)	373,089 (20.06)	1048 (16.14)	657 (12.06)	22,769 (18.34)
Crude RR (95%CI)		0.80 (0.76–0.85)	0.60 (0.55–0.65)	0.91 (0.90–0.93)
Adjusted RR (95%CI)		0.83 (0.77–0.88)	0.63 (0.58–0.68)	0.94 (0.93–0.96)
Severe Maternal Morbidity, n (%)	25,729 (1.38)	226 (3.48)	140 (2.57)	1908 (1.54)
Crude RR (95%CI)		2.51 (2.21–2.87)	1.86 (1.57–2.19)	1.11 (1.06–1.16)
Adjusted RR (95%CI)		2.31 (2.02–2.63)	1.58 (1.34–1.87)	1.00 (0.95–1.04)
Prolonged Maternal Hospital Stay, n (%)	226,275 (12.17)	2090 (32.18)	1295 (23.77)	19,889 (16.02)
Crude RR (95%CI)		2.64 (2.53–2.76)	1.95 (1.85–2.06)	1.32 (1.29–1.33)
Adjusted RR (95%CI)		2.54 (2.44–2.65)	1.81 (1.71–1.91)	1.25 (1.23–1.27)
Preterm Birth, n (%)	93,011 (5.00)	1364 (21)	689 (12.64)	7873 (6.34)
Crude RR (95%CI)		4.20 (3.98–4.43)	2.52 (2.34–2.72)	1.27 (1.24–1.30)
Adjusted RR (95%CI)		3.88 (3.68–4.10)	2.19 (2.03–2.36)	1.15 (1.12–1.18)
Fetal Overgrowth, n (%)	28,614 (1.54)	779 (11.99)	413 (7.58)	5480 (4.41)
Crude RR (95%CI)		7.79 (7.26–8.37)	4.92 (4.47–5.43)	2.86 (2.78–2.95)
Adjusted RR (95%CI)		7.61 (7.09–8.18)	4.71 (4.27–5.19)	2.79 (2.71–2.87)

RR. Relative risk.

## Data Availability

Access to the data is restricted, since the Spanish Ministry of Health requires the investigators to accept the following obligations prior to transferring the data: (1) to treat all information under strict confidentiality conditions; (2) not to use and not to authorize any natural or legal person to use the transferred data other than exclusively for the purposes of the work as reflected in the request; (3) to destroy the file or data provided and all copies made of it once the period of time for which the data are required has elapsed. Requesting access to the data from the Spanish Ministry of Health can be made at https://www.mscbs.gob.es/estadEstudios/estadisticas/estadisticas/estMinisterio/SolicitudCMBDdocs/2018_Formulario_Peticion_Datos_RAE_CMBD.pdf. [Accessed on 9 December 2024]. In any case, we consider that all relevant data are within this paper.

## Supplementary Materials

The following supporting information can be downloaded at https://www.mdpi.com/article/10.3390/jcm13247740/s1, Table S1: ICD 10 Diagnostic and procedure codes used in this investigation; Figure S1: Temporal trends in induced labor among pregnancies in women with type 1, type 2, and gestational diabetes compared with women without diabetes; Figure S2: Temporal trends in cesarean delivery among pregnancies in women with type 1, type 2, and gestational diabetes compared with women without diabetes; Figure S3: Temporal trends in forceps/vacuum extraction among pregnancies in women with type 1, type 2, and gestational diabetes compared with women without diabetes; Figure S4: Temporal trends in episiotomy among pregnancies in women with type 1, type 2. and gestational diabetes compared with women without diabetes; Figure S5: Temporal trends in the prevalence of severe maternal morbidity among pregnancies in women with type 1, type 2, and gestational diabetes compared with women without diabetes; Figure S6: Temporal trends in preterm birth in women with type 1, type 2, and gestational diabetes compared with women without diabetes; Figure S7: Temporal trends in fetal overgrowth among pregnancies in women with type 1, type 2, and gestational diabetes compared with women without diabetes; and Figure S8: Temporal trends in prolonged maternal hospital stay in women with type 1, type 2, and gestational diabetes compared with women without diabetes.
