# Supplementary material for: Pregnancy Outcomes and Maternal Characteristics in Women with Pregestational and Gestational Diabetes: A Population-Based Study in Spain, 2016–2022
Source: J Clin Med. 2024 Dec 18;13(24):7740. doi: 10.3390/jcm13247740 (PMC11679584; doi:10.3390/jcm13247740)
Supplement: Supplementary file 1 [file jcm-13-07740-s001.zip › jcm-3340774-supplementary.pdf]

Table S1. ICD 10 Diagnostic and procedure codes used in this investigation.

Definition	ICD-10-ES
Type 1 diabetes mellitus	E10.x
Type 2 diabetes mellitus	E11.x
Gestational diabetes	O24.4
Fetal overgrowth	P08.0, P08.1, O36.6xxx
Preterm birth	O60. 1
Cesarean delivery	10D00Z0, 10D00Z1, 10D00Z2.
Induced labor	10907ZC
Forceps/vacuum extraction	O66.5<br>10D07Z3, 10D07Z4, 10D07Z5, 10D07Z6,
Episiotomy	0W8NXZZ

Figure S1. Temporal trends in **induced labor** among pregnancies in women with type 1, type 2. and gestational diabetes compared with women without diabetes

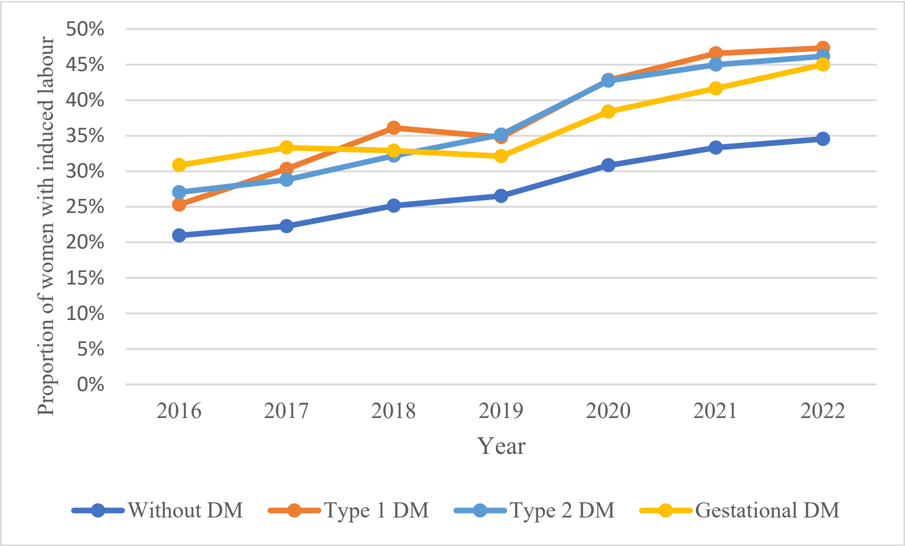

p-value for trend <0.001 in women with type 1 diabetes mellitus, in women with type 2 diabetes mellitus, in women with gestational diabetes and in women without diabetes

Figure S2. Temporal trends in **caesarean delivery** among pregnancies in women with type 1, type 2. and gestational diabetes compared with women without diabetes

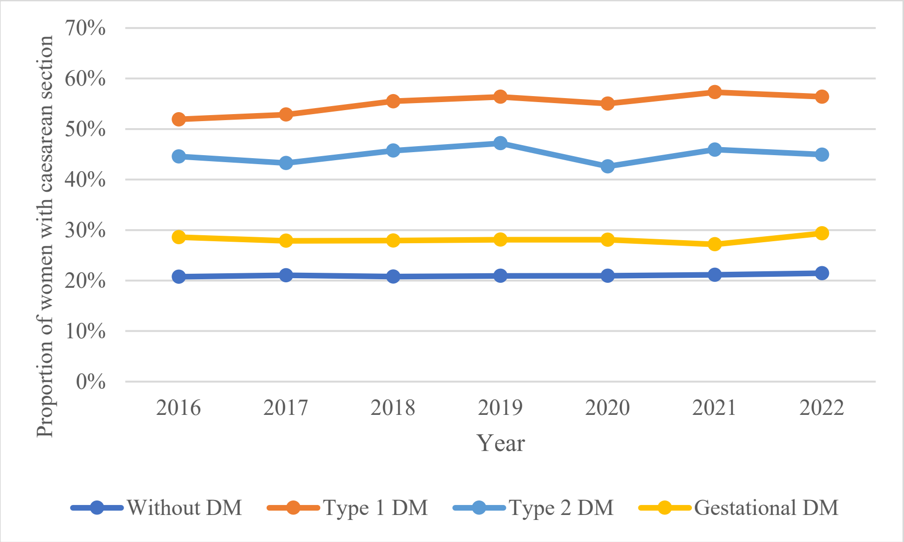

p-value for trend <0.001 in women gestational diabetes

Figure S3. Temporal trends in **Forceps/ Vacuum extraction** among pregnancies in women with type 1, type 2. and gestational diabetes compared with women without diabetes

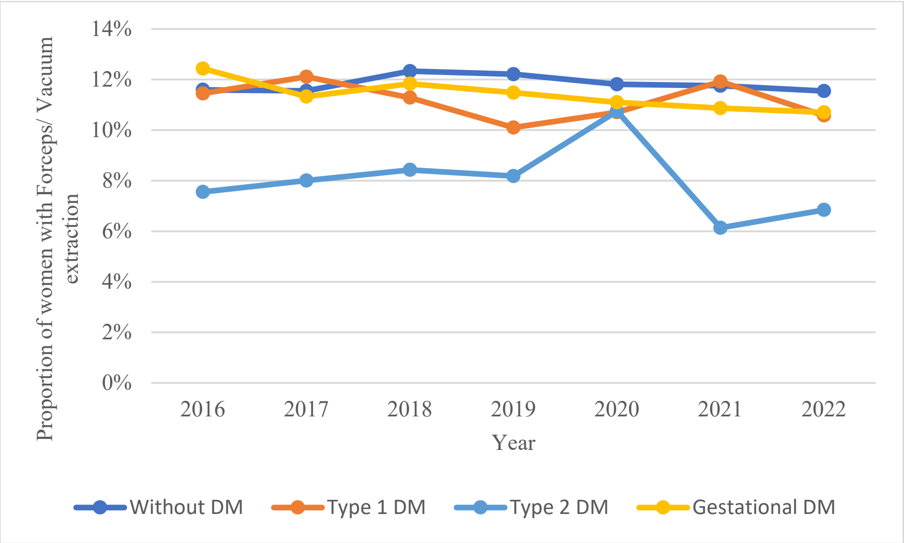

p-value for trend <0.001 in women with type 2 diabetes mellitus and in women with gestational diabetes.

Figure S4. Temporal trends in **Episiotomy** among pregnancies in women with type 1, type 2. and gestational diabetes compared with women without diabetes

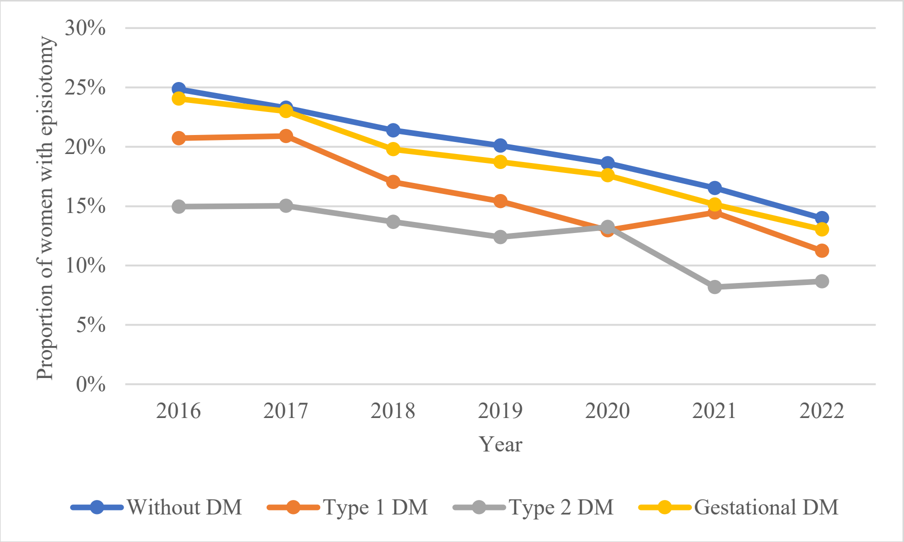

p-value for trend <0.001 in women with type 1 diabetes mellitus, in women with type 2 diabetes mellitus, in women with gestational diabetes and in women without diabetes

Figure S5. Temporal trends in prevalence of **severe maternal morbidity** among pregnancies in women with type 1, type 2 and gestational diabetes compared with women without diabetes.

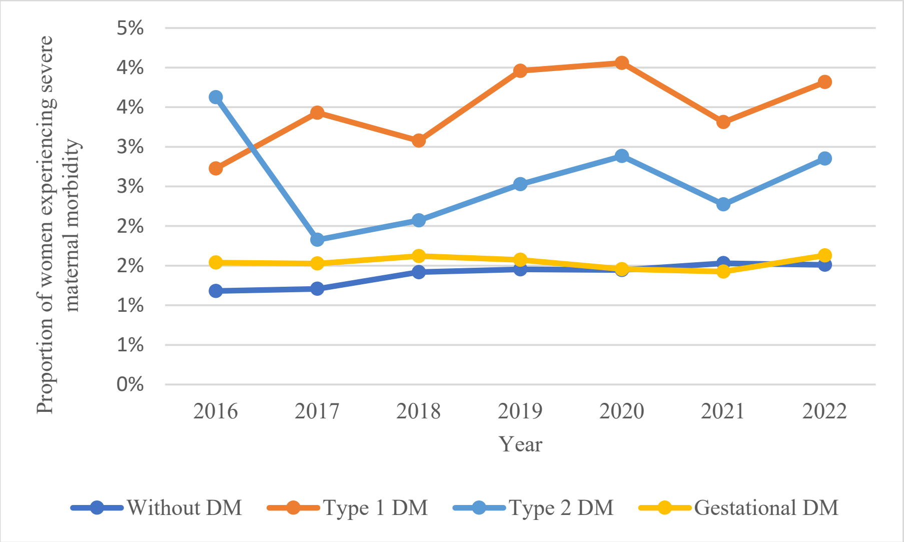

p-value for trend <0.001 in women without diabetes

Figure S6. Temporal trends in **preterm birth** in women with type 1, type 2 and gestational diabetes compared with women without diabetes.

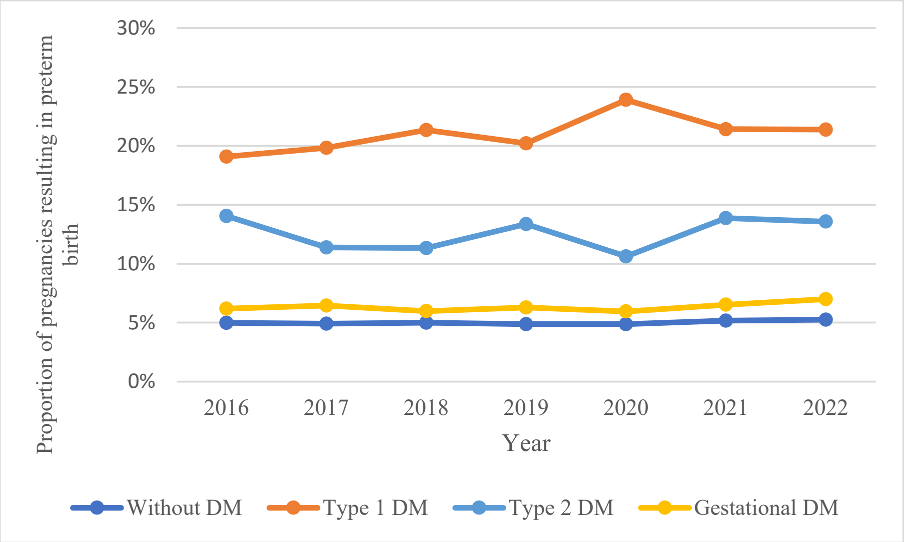

p-value for trend <0.001 in women with gestational diabetes and in women without diabetes.

Figure S7. Temporal trends in **fetal overgrowth** among pregnancies in women with type 1, type 2 and gestational diabetes compared with women without diabetes.

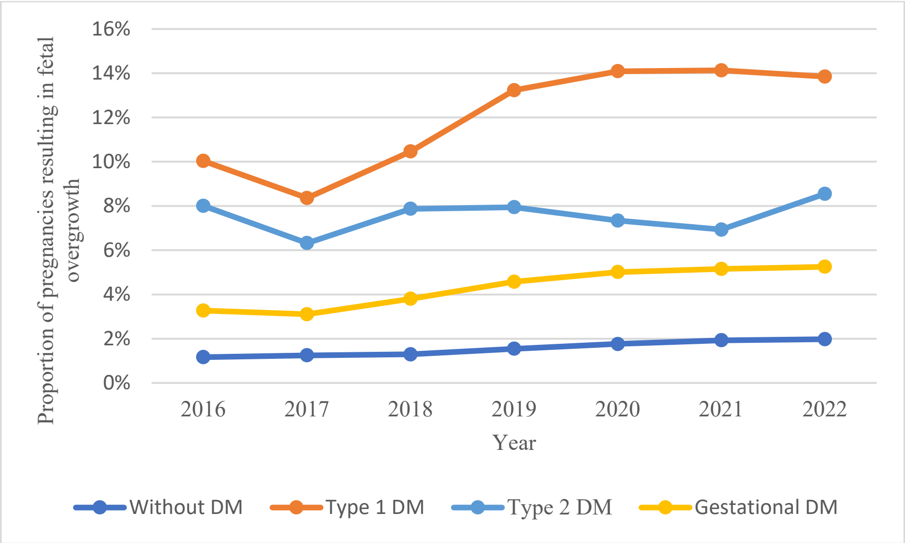

p-value for trend <0.001 in women with type 1 diabetes mellitus, in women with gestational diabetes and in women without diabetes

Figure S8. Temporal trends in **prolonged maternal hospital stay** in women with type 1, type 2 and gestational diabetes compared with women without diabetes.

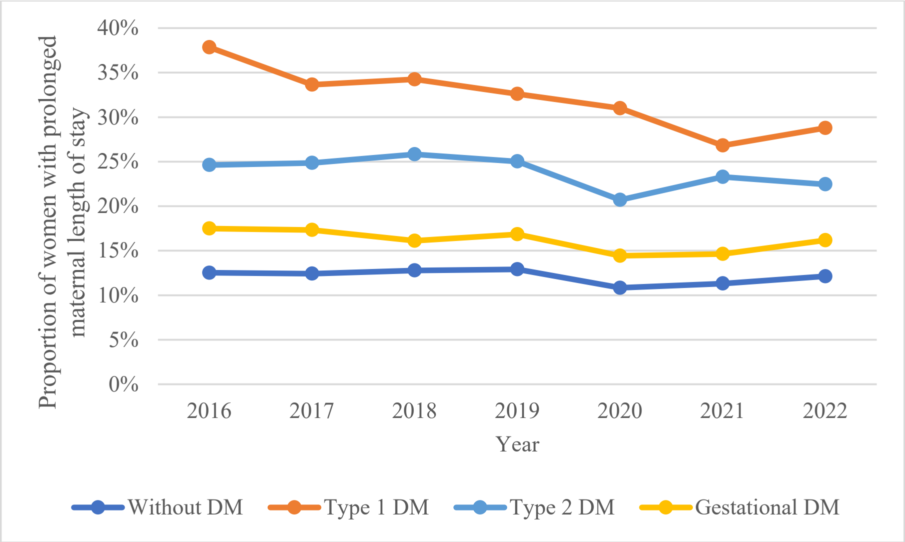

p-value for trend <0.001 in women with type 1 diabetes mellitus, in women with gestational diabetes and in women without diabetes
